# Supplementary material for: Inflammatory and infectious upper respiratory diseases associate with 41 genomic loci and type 2 inflammation
Source: Nat Commun. 2023 Jan 18;14:83. doi: 10.1038/s41467-022-33626-w (PMC9849224; doi:10.1038/s41467-022-33626-w)
Supplement: Supplementary file 1 — Supplementary Information [file 41467_2022_33626_MOESM1_ESM.pdf]

Supplemental material

Contents

Figure S1: Genome-wide association of individual IURDs 2
Figure S2: Genome-wide association of IURD subgroups 3
Figure S3: Genome-wide association of all IURDs 4
Figure S4: Genetic correlation between IURD phenotypes and PheWAS-linked phenotypes 5
Figure S5: IURD genetic correlation with COVID-19 phenotypes. 6
Figure S6: Locus zoom plot of NFKBIZ locus in GWAS of peritonsillar abscess. 7
Table S1: Lead SNPs of genome-wide associated loci in GWAS of sinonasal diseases (n = 25,235) 8
Table S2: Lead SNPs of genome-wide associated loci in GWAS of CISD (n = 19,901) 9
Table S3: Most compelling evidence for each reported locus 10
Table S4: Finngen study approvals 11
Table S5: Biobank access decisions used in this study 12

Figures

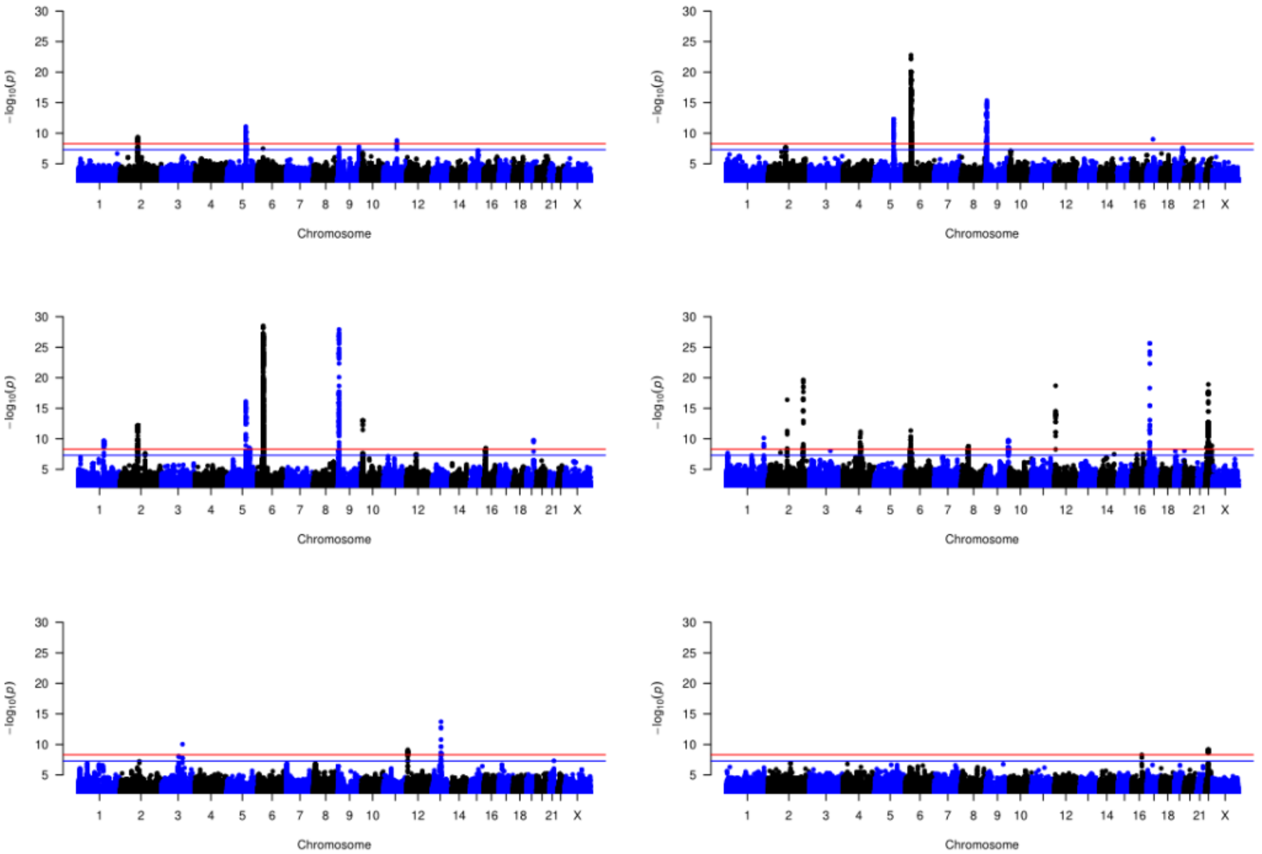

Supplementary Figure 1: Genome-wide association of individual IURDs. Manhattan plots of GWAS of IURD phenotypes against 199,208 controls. The purple line indicates genome-wide significance (GWS; $p < 5e\text{-}8$) and the red line indicates multiple-testing significance ($p < 5e\text{-}9$). A (top left): 8975 cases of VAR link to three MTS loci (six GWS); B (top right): 10,435 cases of CRS distinguish four MTS loci (seven GWS); C (middle left): 3919 cases of NP associate with nine MTS loci (13 GWS); D (middle right): 29,135 cases of CDTA associate with 14 MTS loci (21 GWS); E (bottom left): 4863 cases of PA associate with three MTS loci (five GWS); F (bottom right): 48,687 cases of DPPT associate with one MTS locus (2 GWS).

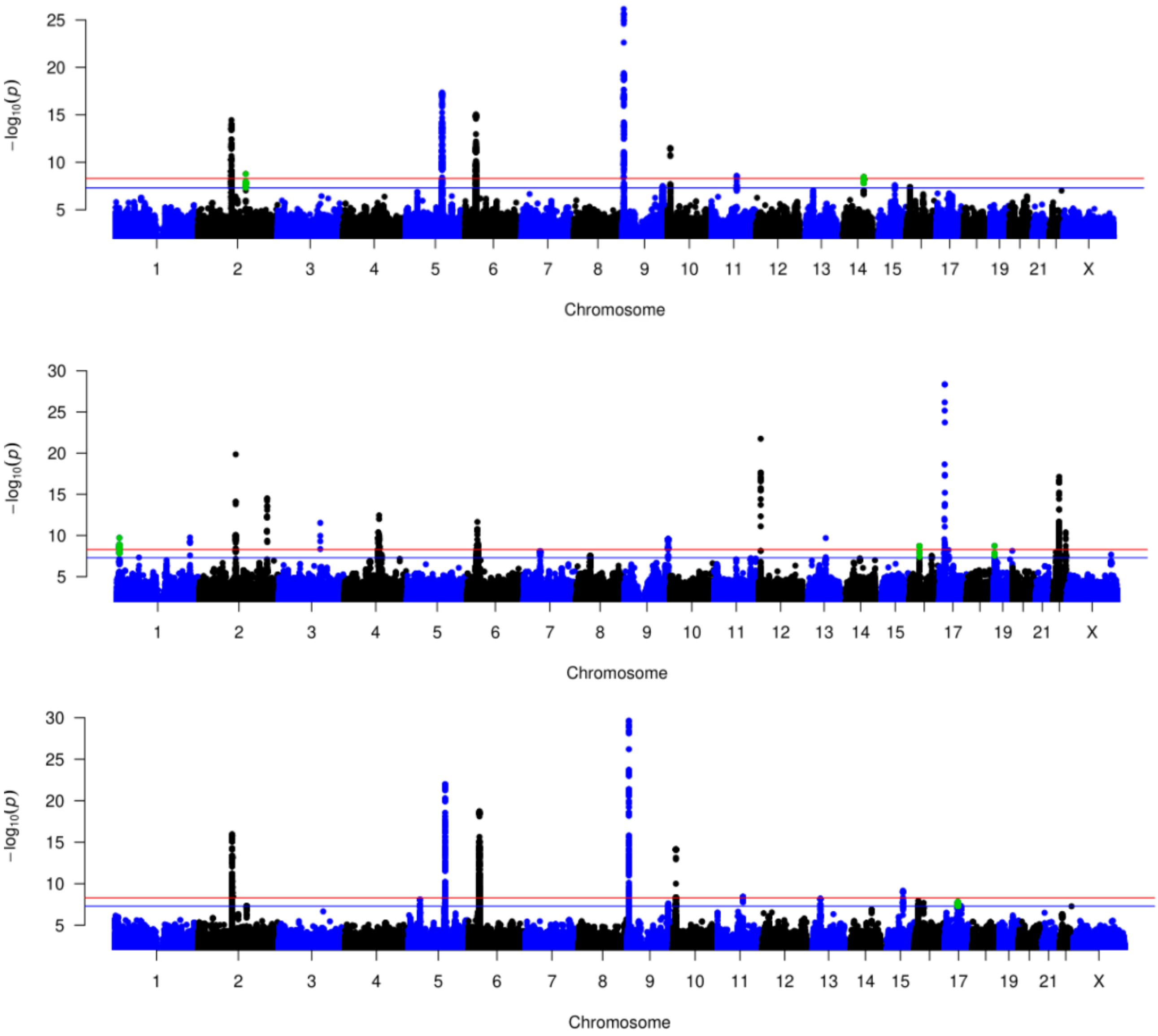

Supplementary Figure 2: Genome-wide association of IURD subgroups. The purple line indicates genome-wide significance (GWS; $p < 5e-8$) and the red line indicates multiple-testing significance (MTS; $p < 5e-9$). A (top): GWAS of sinonasal diseases (n = 25,235) detected eight MTS loci (13 GWS), replicating six loci identified by VAR, CRS, NSD or NP analyses and additionally implicating two genomic regions (2q22.3 and 14q31.1).
B (middle): GWAS of pharyngeal diseases (n = 33,157) detected 16 MTS loci (25 GWS), replicating 13 loci associated with CDTA and PA and distinguishing three additional genomic regions (1p36.23, 16p11.2, and 19p13.3). C (bottom): GWAS of CISD (n = 19,901), a subgroup of sinonasal diseases, associated six non-*HLA* MTS loci (15 GWS), replicating six loci and newly implicating the GWS 17q21.1 locus which also replicated in the UKB. Loci not identified in the specific GWASs are highlighted with green.

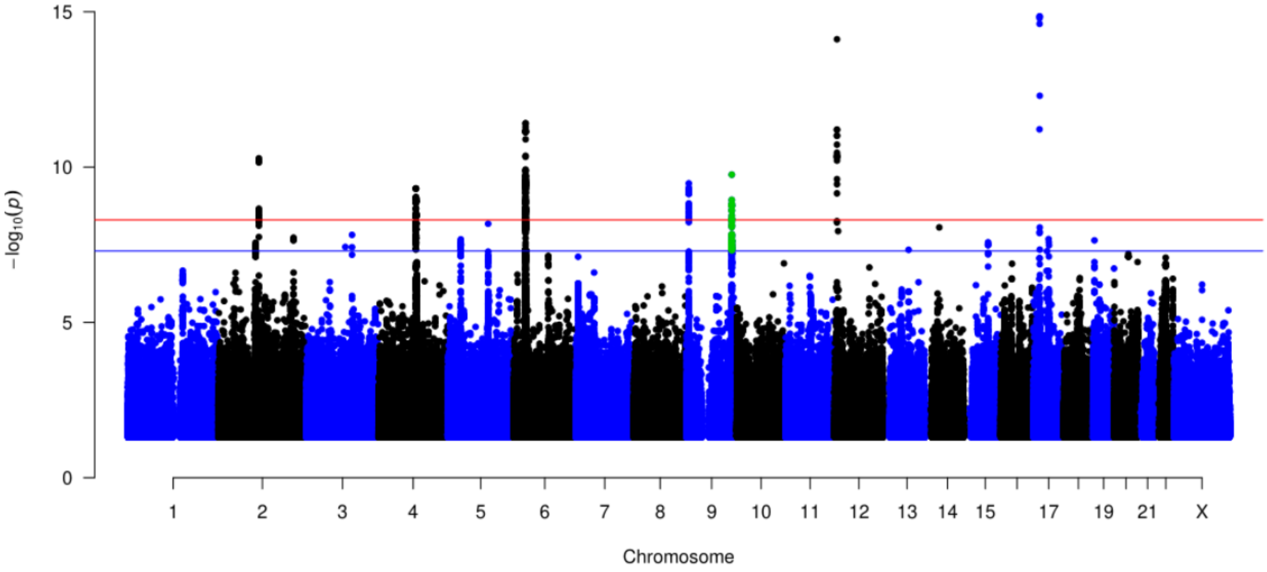

Supplementary Figure 3: Genome-wide association of inflammatory and infectious upper respiratory diseases (IURDs). The purple line indicates genome-wide significance (GWS; p < 5e-8) and the red line indicates multiple-testing significance (MTS; p < 5e-9). GWAS of 61,197 cases of any IURD in the FinnGen study, detecting eight MTS loci (20 GWS) including the novel 9q33.3 locus (green).

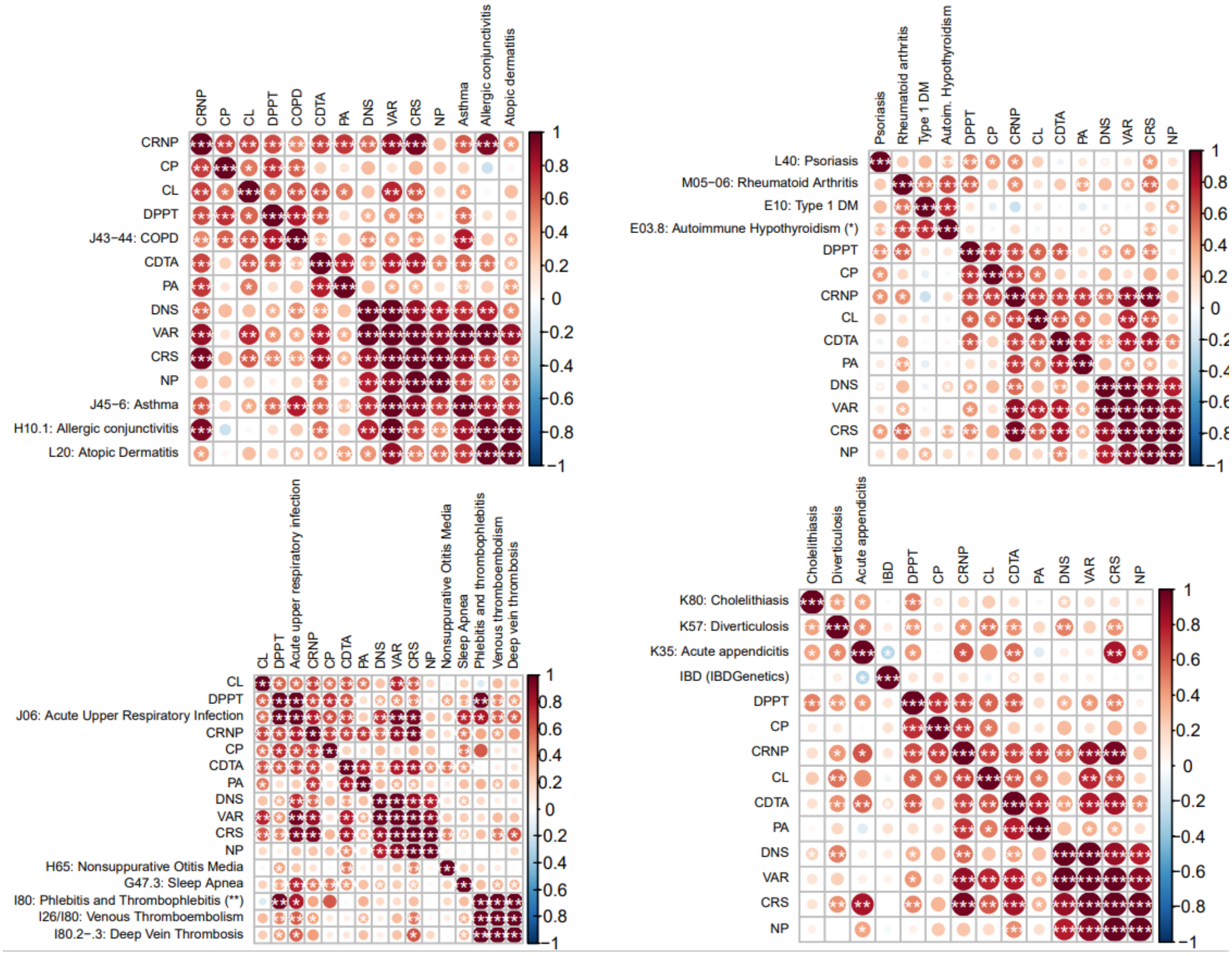

Supplementary Figure 4: Genetic correlation between IURD phenotypes and PheWAS-linked phenotypes. Genetic correlation was measured using LD Score regression. Each plot employs unsupervised hierarchical clustering. **A (top left):** Genetic correlation with allergic diseases and asthma, showing strong clustering with VAR, CRS, and NP. **B (top right):** Genetic correlation with autoimmune disorders, showing wide correlation with rheumatoid arthritis in particular. *Autoimmune hypothyroidism included all hypothyroidism cases, with non-autoimmune causes excluded post hoc. **C (bottom left):** Genetic correlation with other diseases of the upper respiratory tract, and thrombotic disorders, showing distinct clustering of sinus disorders from other chronic IURD infections. **Phlebitis and thrombophlebitis included all I80 cases except I80.2-.3.
D (bottom right): Genetic correlation with inflammatory intestinal disorders. IBD (IBDGenetics) = Inflammatory bowel disease, summary statistics from Liu et al (ref 86). All other endpoints are from the FinnGen database. Inside the matrices: *p < 0.05, **p < 0.005, ***p < 1.9e-4. VAR = Vasomotor and allergic rhinitis; CRNP = Chronic rhinitis, nasopharyngitis and pharyngitis; CRS = Chronic rhinosinusitis; NP = Nasal polyps; NSD = Nasal septal deviation; CDTA = Chronic diseases of tonsils and adenoids; PA = Peritonsillar abscess; CLT = Chronic laryngitis and laryngotracheitis; DPPT = Diseases of pulp and periapical tissues; CP = Chronic periodontitis. P-values were calculated using upper tail chi-square testing (one degree of freedom) from a z-statistic

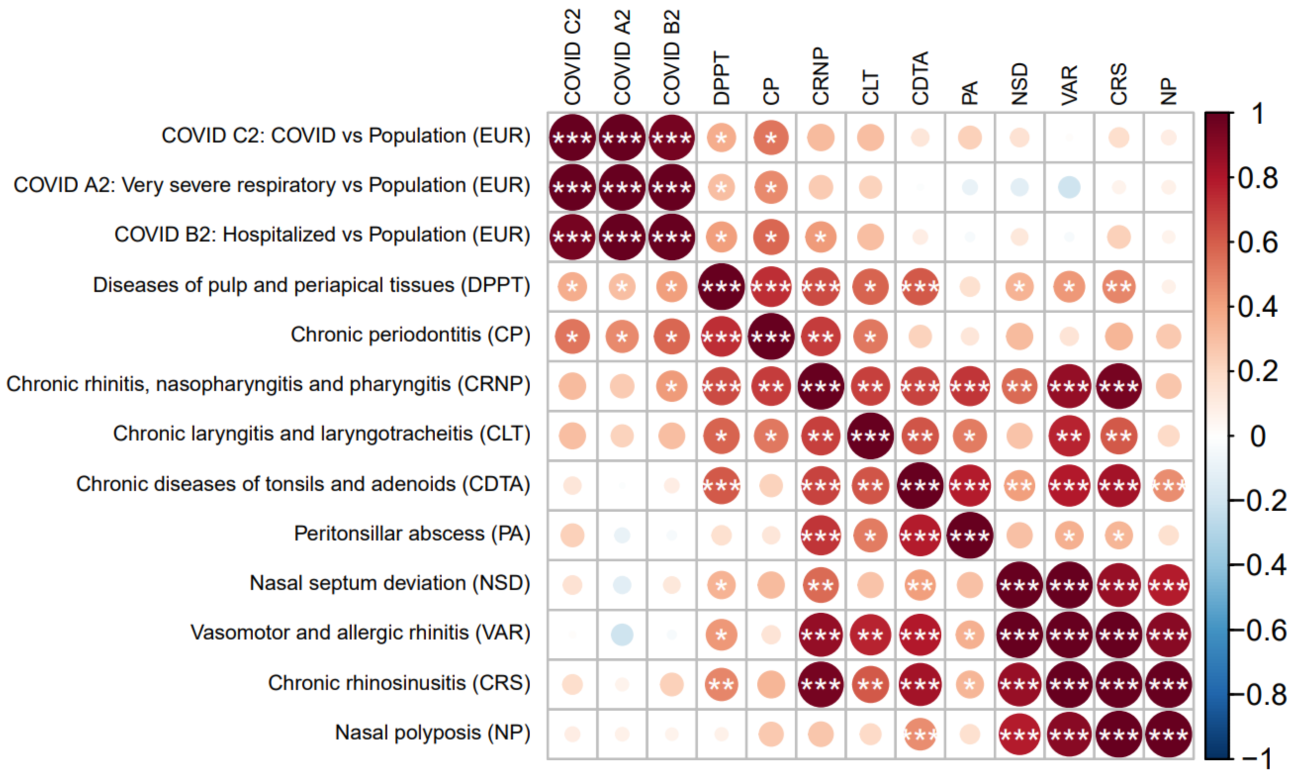

Supplementary Figure 5: IURD genetic correlation with COVID-19 phenotypes. Genetic correlation was measured using LD Score regression. The COVID-19 GWAS summary statistics are from the Host Genetics Initiative (ref. 18). Genetic correlation was observed between COVID-19 endpoints and DPPT, CP, and CRNP. *$p < 0.05$, **$p < 0.005$, ***$p < 1.9e-4$. P-values were calculated using upper tail chi-square testing (one degree of freedom) from a z-statistic.

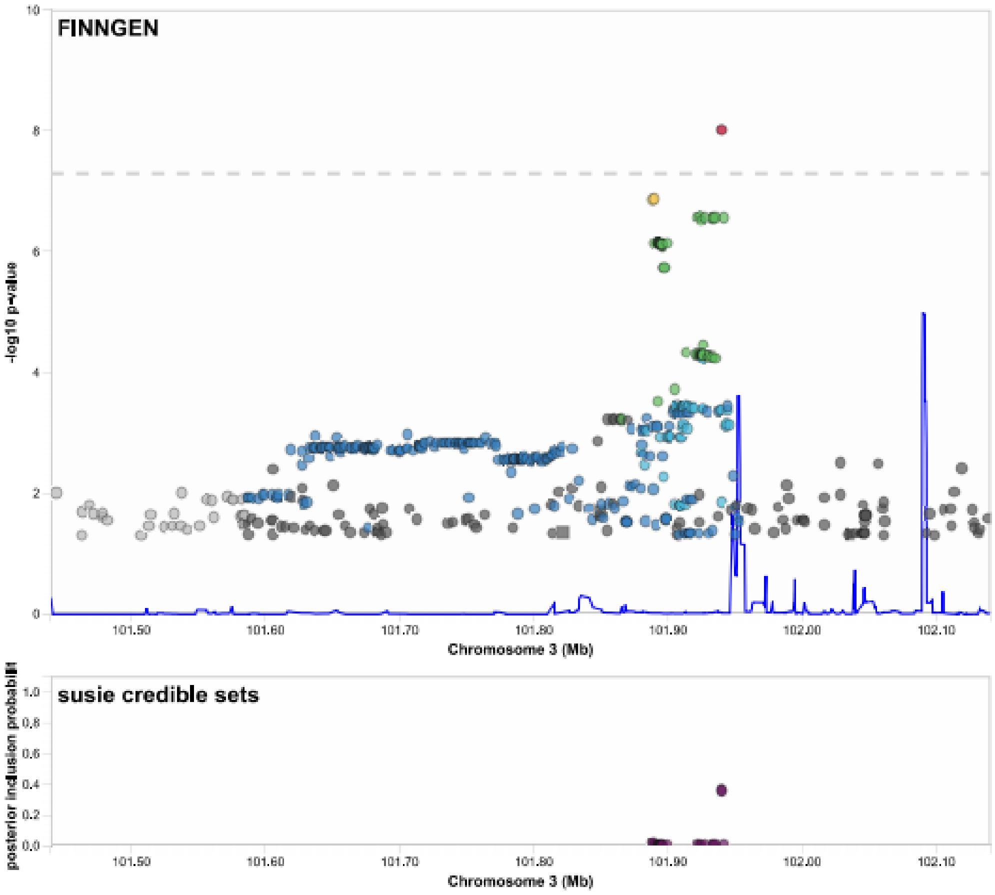

Supplementary Figure 6: Locus zoom plot of *NFKBIZ* locus in GWAS of peritonsillar abscess. Top: p-values of SNPs in the genome-wide association analysis, with lead SNP denoted in purple and color scaling from red to yellow to green to blue in decreasing LD. Blue line is the population recombination rate at different genomic locations. Bottom: SNPs in credible set, with posterior probability on the vertical axis.

Supplementary Table 1: Lead SNPs of genome-wide associated loci in GWAS of sinonasal diseases (n = 25,235). Phenotypes included in this group (allergic sinonasal diseases and DNS) have effect sizes annotated with 95 % CI. Odds ratios (OR) were estimated using logistic regression (Methods). P-values were calculated using upper tail chi-square testing (one degree of freedom) from a t-statistic under a normal approximation. 95 % CI were derived using normal approximation. 'Alleles' denotes the reference / effect alleles. EAF: Effect allele frequency. The HLA locus is annotated for the lead variant according to each phenotype, not the corresponding SNP of the parent GWAS (sinonasal diseases). Asterisks (*) annotate loci not identified in IURD GWAS.

Sinonasal diseases lead variants

					J30,J32,J33,J34.2: Sinonasal diseases (n = 25,235)			
							CISD	J34.2: NSD
BAND	Alleles	Nearest gene	rsid	EAF	OR (95 % CI)	*p*	OR (95 % CI)	OR (95 % CI)
2q12.1	T/A	*IL18R1,IL1RL1*	rs10208293	26.4 %	0.91 (0.89–0.94)	3.58E-15	0.91 (0.88–0.93)	0.95 (0.91–0.99)
*2q22.3	A/G	*ZEB2*	rs16825450	10.3 %	0.90 (0.87–0.94)	1.69E-09	0.90 (0.87–0.94)	0.88 (0.83–0.94)
*5q21.3	G/A	*MAN2A1*	rs7737074	62.8 %	0.94 (0.92–0.97)	3.70E-08	0.94 (0.92–0.97)	0.94 (0.91–0.98)
*5q21.3	A/T	*TMEM232*	rs187769944	2.3 %	1.21 (1.13–1.30)	1.47E-08	1.25 (1.15–1.35)	1.20 (1.06–1.35)
5q22.1	C/G	*WDR36*	rs6884870	30.4 %	1.10 (1.07–1.13)	4.45E-18	1.13 (1.10–1.16)	1.05 (1.01–1.09)
6p21.32	-	*HLA*	-	-	1.11 (1.08–1.14)	9.50E-16	1.14 (1.10–1.18)	0.92 (0.88–0.96)
9p24.1	T/C	*IL33*	rs2095044	76.0 %	0.88 (0.85–0.91)	7.22E-27	0.86 (0.83–0.89)	0.92 (0.88–0.97)
9q33.3	A/G	*NEK6*	rs1107342	40.7 %	1.06 (1.03–1.09)	3.20E-08	1.06 (1.04–1.09)	1.03 (0.99–1.07)
*10p14	G/A	*GATA3*	rs1663680	30.2 %	0.93 (0.90–0.95)	3.34E-12	0.91 (0.88–0.94)	0.95 (0.91–0.99)
*11q13.5	A/G	*EMSY*	rs10160518	43.8 %	1.06 (1.04–1.09)	2.79E-09	1.07 (1.04–1.10)	1.04 (1.00–1.08)
*14q31.1	C/A	*NRXN3*	rs1022434	92.9 %	1.13 (1.08–1.18)	3.47E-09	1.12 (1.07–1.18)	1.14 (1.07–1.23)
15q22.33	C/T	*SMAD3*	rs17293632	26.2 %	1.07 (1.04–1.10)	2.66E-08	1.08 (1.05–1.11)	1.05 (1.01–1.10)
*16p13.13	C/T	*CLEC16A*	rs8064154	31.1 %	0.94 (0.92–0.97)	4.00E-08	0.94 (0.92–0.97)	0.95 (0.92–1.00)

Supplementary Table 2: Lead SNPs of genome-wide associated loci in GWAS of CISD (n = 19,901). Phenotypes included in this group (VAR, CRS and NP) have effect sizes annotated with 95 % CI. Odds ratios (OR) were estimated using logistic regression (Methods). P-values were calculated using upper tail chi-square testing (one degree of freedom) from a t-statistic under a normal approximation. 95 % CI were derived using normal approximation. 'Alleles' denotes the reference / effect alleles. EAF: Effect allele frequency. The HLA locus is annotated for the lead SNP according to each phenotype, not the corresponding SNP of the parent GWAS (CISD). Asterisks (*) annotate loci not identified in IURD or sinonasal disease GWAS.

CISD lead variants

					J30,J32,J33: Chronic inflammatory sinonasal diseases (n = 19,901)				
							J30: VAR	J32: CRS	J33: NP
**Band**	**Allele**	**rsid**	**Nearest gene**	**EAF**	**OR (95 % CI)**	***p***	**OR (95 % CI)**	**OR (95 % CI)**	**OR (95 % CI)**
2q12.1	A/G	rs11690644	*IL18R1,IL1RL1*	15.9 %	0.88 (0.85–0.91)	1.08E-16	0.88 (0.83–0.92)	0.9 (0.86–0.94)	0.88 (0.82–0.94)
2q22.3	T/C	rs1370525	*ZEB2*	8.9 %	0.9 (0.86–0.94)	4.64E-08	0.95 (0.89–1.01)	0.89 (0.84–0.94)	0.79 (0.72–0.86)
5p13.2	G/T	rs4594881	*IL7R*	39.6 %	0.94 (0.91–0.97)	7.87E-09	0.94 (0.90–0.97)	0.95 (0.91–0.98)	0.89 (0.84–0.94)
5q22.1	A/G	rs6884870	*TSLP*	30.4 %	1.13 (1.09–1.16)	1.02E-22	1.1 (1.06–1.15)	1.13 (1.09–1.18)	1.26 (1.19–1.34)
6p21.32	-	-	*HLA*	-	1.14 (1.10–1.18)	1.94E-19	1.1 (1.06–1.14)	1.17 (1.13–1.21)	1.45 (1.36–1.54)
9p24.1	T/C	rs2095044	*IL33*	76.0 %	0.86 (0.83–0.89)	2.27E-30	0.9 (0.86–0.94)	0.87 (0.83–0.90)	0.7 (0.65–0.74)
9q33.3	C/T	rs3758213	*NEK6*	38.2 %	0.94 (0.91–0.96)	2.32E-08	0.91 (0.88–0.95)	0.94 (0.90–0.97)	0.96 (0.91–1.01)
10p14	T/C	rs1663680	*GATA3*	30.2 %	0.91 (0.88–0.94)	7.34E-15	0.92 (0.89–0.96)	0.92 (0.88–0.95)	0.82 (0.78–0.87)
11q13.5	G/A	rs7936323	*EMSY*	41.5 %	1.06 (1.03–1.09)	3.76E-09	1.12 (1.07–1.16)	1.01 (0.97–1.05)	1.05 (0.99–1.11)
*13q14.11	T/C	rs2701859	*FOXO1*	28.7 %	1.08 (1.04–1.11)	6.62E-09	1.07 (1.03–1.12)	1.07 (1.03–1.11)	1.09 (1.03–1.15)
15q22.33	C/T	rs56062135	*SMAD3*	26.2 %	1.08 (1.05–1.11)	6.81E-10	1.1 (1.06–1.15)	1.06 (1.02–1.10)	1.12 (1.05–1.18)
16p13.13	C/T	rs11644510	*CLEC16A*	33.6 %	0.93 (0.90–0.96)	1.13E-08	0.95 (0.91–0.99)	0.92 (0.88–0.96)	0.84 (0.79–0.90)
*16p12.1	G/A	rs74630264	*IL4R*	8.1 %	0.89 (0.85–0.93)	2.02E-08	0.88 (0.82–0.93)	0.91 (0.86–0.97)	0.86 (0.78–0.94)
*17q21.1	A/G	rs3816470	*IKZF3*	56.8 %	0.94 (0.91–0.96)	1.57E-08	0.94 (0.91–0.98)	0.93 (0.90–0.97)	0.9 (0.85–0.95)
*17q21.32	C/T	rs28397663	*PHOSPHO1*	42.6 %	1.06 (1.04–1.09)	4.74E-08	1.06 (1.02–1.10)	1.06 (1.02–1.09)	1.09 (1.03–1.14)

Supplementary Table 3: Most compelling evidence for each reported locus. Reported loci in table 2 were included if genome-wide significant in meta-analysis (”META-ANALYSIS”), multiple-testing significant in FinnGen (”MULTIPLE TESTING”) even if not replicated in UKB, or if a genome-wide significant locus in FinnGen was nominally significant in the UKB analysis (“REPLICATION”). *not previously reported with annotated phenotype

LOCUS	RSID	GENE	PHENOTYPES	VERIFICATION
9q33.3*	rs3758213-T	*NEK6*	VAR	META-ANALYSIS
11q13.5	rs11236795-T	*EMSY*	VAR	META-ANALYSIS
1q21.3*	rs2089081-T	*ARNT*	NP	META-ANALYSIS
2q12.1	rs56117144-C	*IL18RAP*	NP, VAR, CRS	META-ANALYSIS
2q22.3*	rs66484168-G	*ZEB2*	Sinonasal (NP)	META-ANALYSIS
5q22.1	rs34962275-G	*WDR36*	NP, VAR, CRS	META-ANALYSIS
5q31.1a	rs11738827-T	*CDC42SE2*	NP	META-ANALYSIS
5q31.1b	rs56399423-C	*SLC22A4*	NP	META-ANALYSIS
9p24.1	rs2095044-T	*IL33*	NP, VAR, CRS	META-ANALYSIS
10p14a	rs10905284-C	*GATA3*	NP	META-ANALYSIS
10p14b	rs962993-T	*GATA3*	NP, VAR, CRS	META-ANALYSIS
12q13.2	rs705702-G	*RAB5B*	NP	META-ANALYSIS
16p13.13	rs34540843-G	*CLEC16A*	NP	META-ANALYSIS
19q13.2	rs338593-T	*CYP2S1*	NP	META-ANALYSIS
1p36.23	rs12082271-T	*SLC45A1*	CDTA	META-ANALYSIS
2p13.2*	rs35668054-T	*DYSF*	CDTA	META-ANALYSIS
2q33.3	rs189411872-G	*ADAM23*	CDTA	META-ANALYSIS
4q24a	rs4648052-T	*NFKB1*	CDTA	META-ANALYSIS
4q24b	rs5860793-D	*TET2*	CDTA	META-ANALYSIS
8p11.21*	rs73631760-C	*SLC20A2*	CDTA	META-ANALYSIS
9q34.2	rs612169-G	*ABO*	CDTA	META-ANALYSIS
12p13.31	rs10849448-A	*LTBR*	CDTA, PA	META-ANALYSIS
19p13.3*	rs74178437-G	*ZBTB7A*	CDTA	META-ANALYSIS
22q12.2	rs713875-G	*HORMAD2*	CDTA	META-ANALYSIS
3q12.3*	rs1456200-A	*NFKBIZ*	PA	META-ANALYSIS
3q21.2	rs1980080-C	*SLC12A8*	PA, CDTA	META-ANALYSIS
13q21.33	rs9542155-T	*KLHL1*	PA	META-ANALYSIS
15q22.33	rs17293632-T	*SMAD3*	CISD (VAR)	META-ANALYSIS
7p12.2*	rs55935382-A	*IKZF1*	Pharyngeal (CDTA)	META-ANALYSIS
17q12*	rs3744374-A	*GAS2L2*	CRS	MULTIPLE TESTING
1q41	rs12128267-G	*DUSP10*	CDTA	MULTIPLE TESTING
2q13*	rs1045267-A	*MIR4435-2HG*	CDTA	MULTIPLE TESTING
17p11.2	rs573841223-G	*TNFRSB13B*	CDTA	MULTIPLE TESTING
22q11.2*	rs78426131-A	*CCDC188*	CDTA	MULTIPLE TESTING
22q13.33*	rs73181194-A	*PIM3*	CDTA	MULTIPLE TESTING
16p11.2	rs6565189-T	*ITGAL*	Pharyngeal (CDTA)	MULTIPLE TESTING
17q21.1	rs3816470-A	*IKZF3*	CISD (CRS)	REPLICATION
14q31.1	rs1022434-A	*NRXN3*	Sinonasal (NSD)	MULTIPLE TESTING
5p13.2	rs6897932-T	*IL7R*	IURD (NP)	REPLICATION
11q12.2	rs174605-G	*FADS2*	NP (MTAG)	REPLICATION

Supplementary Table 4: Finngen study approvals

**Institution**	**Permit number**
Finnish Institute for Health and Welfare (THL)	THL/2031/6.02.00/2017
Finnish Institute for Health and Welfare (THL)	THL/1101/5.05.00/2017
Finnish Institute for Health and Welfare (THL)	THL/341/6.02.00/2018
Finnish Institute for Health and Welfare (THL)	THL/2222/6.02.00/2018
Finnish Institute for Health and Welfare (THL)	THL/283/6.02.00/2019
Finnish Institute for Health and Welfare (THL)	THL/1721/5.05.00/2019
Finnish Institute for Health and Welfare (THL)	THL/1524/5.05.00/2020
Finnish Institute for Health and Welfare (THL)	THL/2364/14.02/2020
Digital and population data service agency (VRK)	VRK43431/2017-3
Digital and population data service agency (VRK)	VRK/6909/2018-3
Digital and population data service agency (VRK)	VRK/4415/2019-3
the Social Insurance Institution (KELA)	KELA 58/522/2017
the Social Insurance Institution (KELA)	KELA 131/522/2018
the Social Insurance Institution (KELA)	KELA 70/522/2019
the Social Insurance Institution (KELA)	KELA 98/522/2019
the Social Insurance Institution (KELA)	KELA 138/522/2019
the Social Insurance Institution (KELA)	KELA 2/522/2020
the Social Insurance Institution (KELA)	KELA 16/522/2020
Statistics Finland	TK-53-1041-17
Statistics Finland	TK-53-90-20

Supplementary Table 5: Biobank access decisions used in this study

Biobank	Accession number
THL Biobank	BB2017_55
THL Biobank	BB2017_111
THL Biobank	BB2018_19
THL Biobank	BB_2018_34
THL Biobank	BB_2018_67
THL Biobank	BB2018_71
THL Biobank	BB2019_7
THL Biobank	BB2019_8
THL Biobank	BB2019_26
THL Biobank	BB2020_1
Finnish Red Cross Blood Service Biobank	7.12.2017
Helsinki Biobank	HUS/359/2017
Auria Biobank	AB17-5154
Biobank Borealis of Northern Finland	Biobank Borealis of Northern Finland_2017_1013
Biobank of Eastern Finland	1186/2018
Finnish Clinical Biobank Tampere	MH0004
Central Finland Biobank	1-2017
Terveystalo Biobank	STB 2018001
