# Supplementary material for: Inflammatory and infectious upper respiratory diseases associate with 41 genomic loci and type 2 inflammation
Source: Nat Commun. 2023 Jan 18;14:83. doi: 10.1038/s41467-022-33626-w (PMC9849224; doi:10.1038/s41467-022-33626-w)
Supplement: Supplementary file 3 — Editorial Assessment Report [file 41467_2022_33626_MOESM3_ESM.pdf]

nature portfolio

GUIDED OPEN ACCESS

nature genetics | nature communications | communications biology

Contents of this report

- **Manuscript details**: overview of your manuscript and the editorial team.
- **Review synthesis**: summary of the reviewer reports provided by the editors.
- **Editorial recommendation**: personalized evaluation and recommendation from all 3 journals.
- **Annotated reviewer comments**: the referee reports with comments from the editors.
- **Open research evaluation**: advice for adhering to best reproducibility practices.

About the editorial process

Because you selected the **Nature Portfolio Guided Open Access option**, your manuscript was assessed for suitability in three of our titles publishing high-quality work across the spectrum of genetics research: *Nature Genetics, Nature Communications*, and *Communications Biology*. More information about Guided Open Access can be found here.

Collaborative editorial assessment

Your editorial team discussed the manuscript to determine its suitability for the Nature Portfolio Guided OA pilot. Our assessment of your manuscript takes into account several factors, including whether the work meets the **technical standard** of the Nature Portfolio and whether the findings are of **immediate significance** to the readership of at least one of the participating journals in the Nature Portfolio Guided Open Access genetics cluster.

Peer review

Experts were asked to evaluate the following aspects of your manuscript:

- **Novelty** in comparison to prior publications;
- **Likely audience** of researchers in terms of broad fields of study and size;
- **Potential impact** of the study on the immediate or wider research field;
- **Evidence** for the claims and whether additional experiments or analyses could feasibly strengthen the evidence;
- **Methodological detail** and whether the manuscript is reproducible as written;
- Appropriateness of the literature review.

Editorial evaluation of reviews

Your editorial team discussed the potential suitability of your manuscript for each of the participating journals. They then discussed the revisions necessary in order for the work to be published, keeping each journal's specific editorial criteria in mind.

Journals in the Nature portfolio will support authors wishing to transfer their reviews and (where reviewers agree) the reviewers' identities to journals outside of Springer Nature.

If you have any questions about review portability, please contact our editorial office at guidedoa@nature.com.

nature portfolio

GUIDED OPEN ACCESS

nature genetics | nature communications | communications biology

Manuscript details

Tracking number	Submission date	Decision date
GUIDEDOA-21-00165	24 June 2021	9 August 2021

**Title**	Inflammatory and infectious upper respiratory diseases associate with 59 genomic loci that link to type 2 inflammation genes	**Corresponding author**	Aarno Palotie<br>**Affiliation:** Helsinki Institute of Life Science
**Preprint information**	There is a preprint of this manuscript posted at Research Square	**Peer review type**	Single-blind

Editorial assessment team

**Primary editor**	**Wei Li**<br>**Home Journal:** *Nature Genetics*, ORCID: 0000-0002-7885-1775<br>**Email:** wei.li@nature.com
**Editorial team members**	**Ingrid Knarston**, *Nature Communications*, ORCID:<br>**George Inglis**, *Communications Biology*, ORCID: 0000-0002-9069-5242
**About your primary editor**	Wei Li obtained her Ph.D. in Genetics and Genomics from Shanghai Institutes for Biological Sciences, Chinese Academy of Sciences in Bin Han's plant genomics and multi-omics research group. For her postdoctoral work, Wei moved to The Rockefeller University and then Weill Cornell Medical College of Cornell University at Anthony A. Sauve's laboratory to study signaling networks in mammalian metabolic diseases such as diabetes. She joined the Nature Genetics team in 2017.

Editorial assessment and review synthesis

Editor's summary and assessment

The authors studied genome-wide association to eight Inflammatory and infectious Upper Respiratory diseases (IURDs) together and separately in the biobank-scale FinnGen study cohort (release 6). The study sample included 260,405 individuals of all ages, where they focused on cases of specialist-diagnosed IURDs (n = 61,197), including their more specific diagnosis. Using meta-analysis, clustering methods and Bayesian framework models, they investigated the shared and distinct genetic impacts among these disorders and related immune-mediated traits. They detected 59 independent genome-wide significant loci, distinguishing impact on sinonasal or pharyngeal diseases, or both. Fine mapping implicated non-synonymous variants in 16 genes, including 10 linked to immune related diseases. The genetic predispositions to IURDs highlight an underlying genetic structure among several of the IURDs that extends to other immune-mediated traits as well.

While the editors jointly decided to send this manuscript out to review, we recognized that there were a number of IURD-related GWAS studies reported previously. We appreciate the value of analyzing shared genetic contributions of IURDs, though have some concerns about the conceptual advance as well as the degree of biological or epidemiological insights.

Editorial synthesis of reviews

While the Referees find the study of considerable potential interest, they have raised substantial concerns regarding the limitations in the study design, which may affect the conclusions and the robustness of some of the results. They have provided suggestions for including additional analyses and using alternative approaches to strengthen the claims. In addition, the Referees also note that the novelty and biological insights are modest.

In summary, the required revisions should include, but are not limited to, those listed here and detailed in the referee comments:

1. Have robust claim on specific subsets; present combined significance with other population outside Finland and with more stringent significance cutoffs (as highlighted by Referees #1-2).
2. Show replication of already published GWAS on the specific phenotypes (as outlined by Referees #1 &3). How to interpret the replication results especially for loci that failed replication?
3. Use other approaches (as suggested by Referee #2) to identify genetic factors that are shared across the individual disease GWAS.
4. Perform additional analyses to strengthen the conclusions, as noted by Referees #1-3.

When revising the paper for *Nature Communications*, we would ask that you address all points raised by the three reviewers.

Editorial recommendation

nature genetics	Revision not invited	The biological insights and the degree of conceptual advance have not matched the criteria for further consideration at *Nature Genetics.*
nature communications	Major Revisions	For further consideration at *Nature Communications*, we would strongly encourage revising the study to provide clearer conclusions on disease-specific subsets (recommendations provided by Reviewer #1) and placing this work in the context of the wider body of literature. It is also essential to address concerns with significance thresholds (raised by Reviewer #1, #2) and lack of replication (raised by Reviewer #3) in order to provide more robust associations and conclusions.
communications biology	Major Revisions	As noted by the Reviewers, further consideration at *Communications Biology* would require improved statistical analyses, discussion and replication of analogous GWASs for specific phenotypes, and identification of genetic factors that are shared across individual disease GWASs.

Next steps

Recommendation Summary

- **Option 1**: Revise for consideration at *Communications Biology*.
- **Option 2**: Revise for consideration at *Nature Communications*.

See the previous page for details. As noted on page 4, Nature Genetics is not able to consider the work further. If you choose to submit the manuscript via Guided Open Access, only the editors at Nature Communications and Communications will discuss the manuscript.

Revision

If you would like to follow our recommendation, please upload the revised manuscript, along with your point-by-point response to the reviewers' reports and editorial advice **using the link provided in the decision letter**. Should you need assistance with our manuscript tracking system, please contact Adam Lipkin, our Nature Portfolio Guided OA support specialist, at guidedOA@nature.com.

Revision checklist

- Cover letter, stating to which journal you are submitting
- Revised manuscript
- Point-by-point response to reviews
- Updated **Reporting Summary** and **Editorial Policy Checklist**
- Supplementary materials (if applicable)

Submission elsewhere

To a journal outside of Nature Portfolio

If you choose to submit your revised manuscript to a journal at another publisher, we can share the reviews with another journal outside of the Nature Portfolio if requested. You will need to request that the receiving journal office contacts us at guidedOA@nature.com. We have included editorial guidance below in the reviewer reports and open research evaluation to aid in revising the manuscript for publication elsewhere.

nature portfolio

GUIDED OPEN ACCESS

nature genetics · nature communications · communications biology

Annotated reviewer reports

The editors have included some additional comments on specific points raised by the reviewers below, to clarify requirements for publication in the recommended journal(s). However, please note that all points should be addressed in a revision, even if an editor has not specifically commented on them.

Reviewer #1	
**Reviewer #1**	This reviewer has not chosen to waive anonymity. The reviewer's identity can only be shared with representatives of an established journal editorial office.
**Reviewer #1 expertise**	statistical genetics, epidemiology
**Editor's comments about this review**	This reviewer has provided insightful feedback regarding the study design and the strength of the claims. They also highlight the need to proofread the manuscript for clarity, and provide a useful framework for the study to make it easier for a reader to logically follow the data.<br>All of these points should be addressed for publication at *Nature Communications.*

Reviewer #1 comments	
**Overview**	Saarentaus and colleagues performed a genome wide association of combined set of infectious upper respiratory diseases (IURDs) in Finland. They claimed associations of variants with IURDs at 59 loci. Whereas it is clear that this represents a large amount of work, the current presentation has major limitations. First it is somewhat unclear what is really claimed, the combined set or the subsets of diseases. Second through the manuscript it is unclear what is novel and what is new; the reader has to arrive to the discussion to see that clearly articulated. I would have found easier to take specifically homogenic unit of disease (i.e., the subset in the current), perform an international meta -analysis with foreign public set such as UKB (UK biobank) and/or BBJ (biobank japan). Then have as robust association as possible, i.e. choose more stringent cutoff than 5E-8, a cutoff that have been proposed a long time ago under different settings (i.e., population, number of markers).

Editorial comments:

For *Nature Communications*, we would also like to see clear justification of significance threshold used and potential exploration of a more robust cut-off.

The point about the cutoff was also raised by Reviewer #2, please justify the use of a 5E-8 cutoff, or update your analyses with a more appropriate threshold. This point is important for publication in either of the 2 journals.

Specific comments

#	Reviewer comment	Editorial comment
**1**	I understand the work of the authors which is somewhat methodological when grouping all the IURDs phenotypes, but I feel that this demonstration somewhat limits the conclusions and the robustness of the results. If the authors succeed to have a clearer presentation including replication and combined analysis with abroad. A large number of the results presented here might be false positive, since 1)the cutoff used 5E-8 had been set under different circumstances, population and number of markers 2) Many of the claimed association are less than an order of magnitude from the lenient threshold 3) most of them 24 out of 41 do not replicate nominally in their foreign group.	
**2**	Additionally, one would expect that the authors attempt replication of all the reported variants in the literature for the phenotypes they study, in order to assess how comparable their groups are with the one used for previously reported associations. For example, a variant in *ALOX15* has been reported to confer a strong and significant protection against NP and CRS. It is somewhat unexpected not to see it mentioned in this manuscript.	
**3**	One of the main issue with this manuscript is the phenotype. I understand the angle the authors have taken: starting with a combination of phenotypes in Finland. But I truly am of the opinion that it makes it very unclear what is really claimed.	
**4**	I would strongly suggest to start with specific phenotypes, performing meta-analysis with other population than Finland and with more stringent significance cutoffs to then arrive to a set of robust associations that they can then assess in the other subsets. In the UK biobank some of these diagnosis have to be derived from the GP sets in addition to hospital diagnosis.	Please carefully proofread the manuscript for clarity. We also encourage you to follow this framework to improve the flow and readability of the manuscript.<br><br>For *Nature Communications*, we would strongly encourage revising the study to provide clearer conclusions and address

		issues with significance thresholds in order to provide more robust associations.
**5**	Also the authors might realize that a lot of these diagnosis are differential diagnosis for each other's, and that a large number of the patients probably overlap between subsets.	
**6**	Personally, I recommend to focus on the strong signal, choose more significant cutoffs. And present the results that are specific on the subsets, once combined with public datasets.	
**7**	As mentioned in different places above, the cutoff for statistical significance of 5E-8 has been proposed many years back at the time where sample size and number of markers tested where different. From the attempt of replication currently displayed it is possible that a large fraction of the association are not true positive.<br>If they were, the ones very close to the cutoff are likely suffering from winner's curse, i.e. an inflation of effect.	
**8**	Using meta-analysis with foreign data should be performed when available.	
**9**	Again, I would rather have claims specific to the subsets that are robust presented first, with combined significance with the foreign groups.	
**10**	I would present replications of all previous reported markers for these phenotypes.	

**Reviewer #2**	
**Reviewer #2**	This reviewer has not chosen to waive anonymity. The reviewer's identity can only be shared with representatives of an established journal editorial office.
**Reviewer #2 expertise**<br>Summarised	human genetics, epidemiology

by the editor	
**Editor's comments about this review**	This reviewer has provided insightful feedback regarding the approaches used in the analyses and the potential impact. This reviewer also highlights the limited reproducibility of the current work, which should be improved for further consideration. All of these points should be addressed for further consideration at *Nature Communications* and *Communications Biology*.

Reviewer #2 comments

**Overview**	The manuscript by Saarentaus and colleagues describes a GWAS of inflammatory and infectious upper respiratory disease (IURD) in ~260k FinnGen study participants. They identify 59 genome-wide significant loci associated with this heterogeneous disease definition and/or component diseases of the umbrella term. I have a few comments for consideration by the authors:

Specific comments

#	Reviewer comment	Editorial comment
1	Identifying loci by lumping together different diseases into a single 'mega case' group is a reasonable approach but I wonder if there are better alternatives. Have the authors explored using Genomic SEM (or similar approaches) to identify genetic factors that are shared across the individual disease GWAS?	
2	The authors have effectively run 12 GWASs but corrected for only one (P<5x10-8) from what I can see. They may want to justify this approach.	Given that concerns about significance thresholds were also raised by Reviewer #1, we would ask that you provide clear justification for this cutoff, or update the analyses with a more appropriate threshold.
3	"Lead variants of the 58 non-HLA GWS loci were associated with 2,861 endpoints in FinnGen" – I suspect this number would be substantially lower if co-localization analyses were run. Presumably it's possible that many of these associations are in "LD shadows" of other signals?	For publication at *Nature Communications*, we would like you to address this concern with additional colocalisation analyses.
4	"We then compared SNPs with expression data from GTEx v8 and DICE, which showed change in the expression of a	

	total of 264 genes in all 58 non-HLA loci in total" – this statement really highlights the limitations of using tools like FUMA to assign causal genes to GWAS hits. The mapping of non-synonymous variants appeared methodologically robust, and I'd suggest the authors apply a similarly conservative approach to eQTL mapping. At a minimum the authors need to ensure they use eQTL integration approaches that reduce spurious associations due to more coincidental LD overlaps.	
5	**Impact**<br><br>Personally I feel this manuscript lacks the novel biological or epidemiological insights that warrant publication in journal such as *Nature Genetics*. A more incremental but rigorous study of this type may be better suited to *Communications Biology* or *Nature Communications*.	
6	**Reproducibility**<br><br>From what I can see it is not possible for anyone outside of FinnGen to access individual level data and reproduce these results. This is a shame given the precedent set by studies such as UK Biobank.	Please carefully review the policies in the Open Research Evaluation to improve the reproducibility of these results.

Reviewer #3	
**Reviewer #3**	This reviewer has not chosen to waive anonymity. The reviewer's identity can only be shared with representatives of an established journal editorial office.
**Reviewer #3 expertise**<br><br>Summarised by the editor	statistical genetics
**Editor's comments about this review**	This reviewer has provided an overall positive assessment of the paper, but please see major concerns #1 and #2. Altogether, they have provided several important points that should be discussed in a revised manuscript for further consideration at *Nature Communications* or *Communications Biology*.

Reviewer #3 comments

**Overview**	The authors performed genome-wide association studies of a series of inflammatory and infectious upper respiratory diseases and identified 59 loci associated with one or more of these diseases, of which 23 are novel. They identified a high degree of sharing between many IURD subtypes, as well as with other related phenotypes such as asthma and allergies. The analyses are appropriate and well-performed and the paper is well-written. I have some questions and concerns that I hope the authors will be able to address:

Specific comments

#	Reviewer comment	Editorial comment
**1**	On page 3, several previous genetic studies of IURDs are mentioned, but the authors do not seem to place their new results in the context of these studies. For instance, when compared to Kristjansson et al 2019, the current manuscript has twice the number of CRS cases and a similar number of NP cases, yet did not report associations at the loss-of-function variant in *ALOX15*. I would be curious to know whether these and other variants reported in the literature also showed associations in FinnGen, and if not, whether aspects of study design (e.g. case definition) or differences in LD patterns can lead to certain loci not being detected.	Appropriate comparison of these results with the literature would be necessary for further consideration at *Communications Biology*.<br><br>For publication at *Nature Communications*, we would like to see a more comprehensive discussion placing this study in the context of the existing literature and commenting on differences underlying the diverging associations found.
**2a**	With regards to the replication work in UKB:<br><br>Page 7: "We found that the effect size differed (p < 0.05) only for the CRS association to the locus 5q22.1 (near WDR36) and the NP association to the locus 1q211.3 (near *ARNT*)."<br><br>In Table S6, it looks like the *GAS2L2/TAF15* association with CRS was also not replicated and has a significant effect size difference in UKB.	
**2b**	In general, how should we interpret loci that failed replication, despite having over 95% power to do so at alpha=0.05? Could it be that some of these diseases that occurred earlier in life are not well-captured in the UKB's cohort of middle-aged participants? Is there	For publication at *Nature Communications*, we would like you to address the concerns in 2b and 2c with addition analyses and discussion, providing clearer

	any evidence of different LD patterns between Finnish and UK population at some of the non-replicating loci?	recommendations of how to interpret these results.
**2c**	Similarly, in UKB, the CDTA phenotype had the smallest effective sample size, while in FinnGen, this phenotype had the largest. Is this a consequence of diseases that occur earlier in life not being as well captured in UKB? If that is the case, how should we interpret the replication results in UKB more generally?	
**3**	In the PheWAS analysis of lead SNPs, Sup table 7 shows 462 significant variant-phenotype pairs. How many of these pairs are actually being most likely driven by the same causal variant versus being driven by LD with another causal variant?	
**4**	Several variants in Table 4 appear to have allele frequencies that are inconsistent with other tables. For instance, rs189411872 has frequency 1.3% in Table S4, but 66% in Table S3.	
**5**	In Table 3, please include the posterior probability of causality for each variant to help quantify the likelihood that the variant is causal. It also appears that rs11557467 and rs2305479 are two missense variants in different genes but belong to the same credible set? Please clarify.	
**6**	Page 7. "Lead variants of the 58 non-HLA GWS loci were associated with 2,861 endpoints in FinnGen". The phrasing here is ambiguous. On first reading I thought this meant that the 58 loci showed significant associations with 2861 other phenotypes, but I think this number refers to the total number of phenotypes tested?	
**7**	Table S4. MAF column should be renamed EAF since many frequencies are > 0.5	
**8**	Table S7. Are the betas with respect to the same ref/effect alleles given in Tables S1-S3? Please clarify	
**9**	"SNP" is used throughout the paper even though many of the lead variants at significant loci are indels.	

Open research evaluation

Data availability

Data Availability statement

Thank you for including a Data Availability statement. We noticed that not all datasets reported in the paper are included in this statement. The data availability statement must make the conditions of access to the “minimum dataset” that are necessary to interpret, verify and extend the research in the article, transparent to readers. More information about our data availability policy can be found here: https://www.nature.com/nature-portfolio/editorial-policies/reporting-standards#availability-of-data

See here for more information about formatting your Data Availability Statement: http://www.springernature.com/gp/authors/research-data-policy/data-availability-statements/12330880

Data availability: This journal strongly supports public availability of data and custom code associated with the paper in a persistent repository where they can be freely and enduringly accessed or as a supplementary data file when no appropriate repository is available. If data and code can only be shared on request, please explain why in your data Availability Statement, and also in the correspondence with your editor. For more information, please refer to https://www.nature.com/nature-research/editorial-policies/reporting-standards#availability-of-data.

Please ensure that datasets deposited in public repositories are now publicly accessible, and that accession codes or DOI are provided in the "Data Availability" section. As long as these datasets are not public, we cannot proceed with the acceptance of your paper. For data that have been obtained from publicly available sources, please provide a URL and the specific data product name in the data availability statement. Data with a DOI should be further cited in the methods reference section.

Mandatory data deposition

For your genome-wide association study, submission of the summary statistics to a community-endorsed, public repository is mandatory for publication in a Nature Portfolio journal and is best practice for publication in any venue. Accession numbers must be provided in the paper. For this data type, we recommend submission to the NHGRI-EBI GWAS Catalog: https://www.ebi.ac.uk/gwas/

For more information on mandatory data deposition policies at the Nature Portfolio, please visit http://www.nature.com/authors/policies/availability.html#data

For an up-to-date list of approved repositories for each mandatory data type, please visit https://www.springernature.com/gp/authors/research-data-policy/repositories/12327124

Data citation

Thank you for depositing your dataset in a public repository. In addition to providing the link within the Data Availability statement, we ask that you also cite the dataset in the main reference list. Citing and referencing data in publications supports reproducible research, by increasing the transparency and provenance tracking of data generated or analysed during research.

Citing data formally in reference lists also helps facilitate the tracking of data reuse and may help assign credit for individuals' contributions to research. A number of Springer Nature imprints are signatories of the Joint Declaration on Data Citation Principles, which stress the importance of data resources in scientific communication.

Code availability and citation

Please include a statement under the heading "Code Availability", indicating whether and how the custom code/software reported in your study can be accessed, including any restrictions to access. This section should also include information on the versions of any software used, if relevant, and any specific variables or parameters used to generate, test, or process the current dataset. Code availability statements should be provided as a separate section after the Data Availability section.

Upon publication, Nature Portfolio journals consider it best practice to release custom computer code in a way that allows readers to repeat the published results. Code should be deposited in a DOI-minting repository such as Zenodo, Gigantum or Code Ocean and cited in the reference list following the guidelines described in our policy pages (see link below). Authors are encouraged to manage subsequent code versions and to use a license approved by the open source initiative.

Full details about how the code can be accessed and any restrictions must be described in the Code Availability statement.

See here for more information about our code availability policies: https://www.nature.com/nature-portfolio/editorial-policies/reporting-standards#availability-of-computer-code

We also provide a Code and Software submission checklist that you may find useful: https://www.nature.com/documents/nr-software-policy.pdf

Please note: because of advanced features used in this form, you must use Adobe Reader to open the documents and fill it out.

Ethics

Please provide a 'Competing interests' statement using one of the following standard sentences:

1. The authors declare the following competing interests: [specify competing interests]
2. The authors declare no competing interests.

See our competing interests policy for further information: https://www.nature.com/nature-research/editorial-policies/competing-interests

Reporting and reproducibility

Reproducibility: Please state in the legends how many times each experiment was repeated independently with similar results. This is needed for all experiments, but is particularly important wherever results from representative experiments (such as micrographs) are shown. If space in the legends is limiting, this information can be included in a section titled “Statistics and Reproducibility” in the methods section.

Statistics

Statistics: Wherever statistics have been derived (e.g. error bars, box plots, statistical significance) the legend needs to provide and define the n number (i.e. the sample size used to derive statistics) as a precise value (not a range), using the wording “n=X biologically independent samples/animals/cells/independent experiments/n= X cells examined over Y independent experiments” etc. as applicable.

Legends requiring revision:

Please note that this information is missing in the legends of figures 3b, d; 4b, d.

Statistics such as error bars, significance and p values cannot be derived from n<3 and must be removed from all such cases.

We strongly discourage deriving statistics from technical replicates, unless there is a clear scientific justification for why providing this information is important. Conflating technical and biological variability, e.g., by pooling technically replicates samples across independent experiments is strongly discouraged. (For examples of expected description of statistics in figure legends, please see the following https://www.nature.com/articles/s41467-019-11636-5 or https://www.nature.com/articles/s41467-019-11510-4).

All error bars need to be defined in the legends (e.g. SD, SEM) together with a measure of centre (e.g. mean, median). For example, the legends should state something along the lines of “Data are presented as mean values +/- SEM” as appropriate.

All box plots need to be defined in the legends in terms of minima, maxima, centre, bounds of box and whiskers and percentile.

Legends requiring revision:

1. Please note that the error bars need to be defined in the legends of figures 3d and 4d.
2. Please note that the measure of centre for the error bars needs to be defined in the legends of figures 3b and 4b.

The figure legends must indicate the statistical test used. Where appropriate, please indicate in the figure legends whether the statistical tests were one-sided or two-sided and whether adjustments were made for multiple comparisons.

For null hypothesis testing, please indicate the test statistic (e.g. F, t, r) with confidence intervals, effect sizes, degrees of freedom and P values noted.

Please provide the test results (e.g. P values) as exact values whenever possible and with confidence intervals noted.

Legends requiring revision:

1. Please indicate the statistical test used for data analysis and where appropriate, please specify whether it was one-sided or two-sided and whether adjustments were made for multiple comparisons, in the legends of figure 1a; tables 2; 3; 4; supplementary figures 1; 2a-c; 3a-f; 4a-d; 5; 6 (1st panel) and supplementary tables 1; 2; 3; 4; 5; 6; 7; 8; 9; 10.
2. Please note that the exact p value should be provided, when possible, in the legends of figure 1a and supplementary figures 4a-d; 5.

Data presentation

When choosing a color scheme please consider how it will display in black and white (if printed), and to users with color blindness. Please consider distinguishing data series using line patterns rather than colors, or using optimized color palettes such as those found at https://www.nature.com/articles/nmeth.1618

The use of colored axes and labels should be avoided. Please avoid the use of red/green color contrasts, as these may be difficult to interpret for colorblind readers.

Data presentation: Please ensure that data presented in a plot, chart or other visual representation format shows data distribution clearly (e.g. dot plots, box-and-whisker plots). When using bar charts, please overlay the corresponding data points (as dot plots) whenever possible and always for n ≤ 10. (Please see the following editorial for the rationale behind this request and an example https://www.nature.com/articles/s41551-017-0079).

Language editing

Other notes

We have included as an attachment to the decision letter a version of your Reporting Summary with a few notes. This is mainly for your information, but we hope it is helpful when preparing your revised manuscript. If you decide to resubmit the manuscript for further consideration, please be sure to include an updated Reporting Summary.

Additional Notes

- Please note that the individual figure panels are not labelled as ‘a, b, etc.’ for figures 1; 3; 4 and supplementary figures 2; 3; 4. Please rectify these in the figure panels.
- Please note that the supplementary figure 6 is incorrectly labelled as ‘7’ in the supplementary dataset 1. Please rectify this.
