# Supplementary material for: Inflammatory and infectious upper respiratory diseases associate with 41 genomic loci and type 2 inflammation
Source: Nat Commun. 2023 Jan 18;14:83. doi: 10.1038/s41467-022-33626-w (PMC9849224; doi:10.1038/s41467-022-33626-w)
Supplement: Supplementary file 4 — Description of Additional Supplementary Files [file 41467_2022_33626_MOESM4_ESM.docx]

# Supplementary Data Legends

## Supplementary Data 1

Lead variant of genome-wide significant loci in GWAS of IURD in FinnGen, and replication of variant (or if missing in UKB, other GWS variant with LD >0.1 if available). Reference variant is chosen based on af>50 % in FinnGen. Heterogeneity tested with direct z-test between beta in FinnGen and UKB (methods); pz < 0.05 was considered heterogeneous. 'Codirectional' denotes if beta signs are the same or not. Meta-analysis was conducted using inverse-variance weighted meta-analysis. Confidence intervals are calculated using normal approximation. P-values are calculated from upper tail chi-square testing (one degree of freedom). *Probability of null hypothesis rejection in the replication analysis, when the true effect is that observed in FinnGen at alpha = 0.05

## Supplementary Data 2

Lead SNPs of genome-wide associated loci in GWAS of pharyngeal diseases (n = 33,157). Phenotypes included in this group (CDTA and PA) have effect sizes annotated with 95 % CI. ‘Alleles’ denotes the reference / effect alleles. EAF: Effect allele frequency. The HLA locus is annotated for each SNP according to the lowest p-value in the phenotype locus, not the corresponding SNP of the parent GWAS (pharyngeal diseases). Odds ratios (OR) were estimated using logistic regression (Methods). P-values were calculated using upper tail chi-square testing (one degree of freedom) from a t-statistic under a normal approximation. 95 % CI were derived using normal approximation. Asterisks (*) annotate loci not identified in CDTA or PA GWASs

## Supplementary Data 3

Lead SNPs of Genome-wide associated loci in GWAS of IURD (n = 61,197). Phenotypes (sinonasal diseases, CRNP, pharyngeal diseases and CL) have effect sizes annotated with 95 % CI. ‘Alleles’ denotes the reference / effect alleles. EAF: Effect allele frequency. The HLA locus is annotated for the lead SNP according to each phenotype, not the corresponding SNP of the parent GWAS (IURD). Odds ratios (OR) were estimated using logistic regression (Methods). P-values were calculated using upper tail chi-square testing (one degree of freedom) from a t-statistic under a normal approximation. 95 % CI were derived using normal approximation. *Replicated locus not observed in phenotype-specific GWASs (including sinonasal or pharyngeal disease GWAS)

## Supplementary Data 4

Lead variants of genome-wide significant (p<5e-8) loci in MTAG analysis. Ref and Alt are set according to default effect allele assignment in the FinnGen panel. Due to the reported inflation of loci detected through MTAG, new loci were only considered likely true if replicated in the UKB analyses (UKB OR and UKB pval). *loci not observed in FinnGen single phenotype or cross-trait GWASs

## Supplementary Data 5

Association of lead variants of genome-wide significant loci in any IURD, and impact across IURDs. Effect allele is default variant for FinnGen panel. Lead variant is chosen based 'top-down', meaning cross-trait analyses are favored above phenotype-specific analyses (e.g. CISD rather than NP, IURD rather than pharyngeal, etc.).

## Supplementary Data 6

Impact of previously reported loci in single-phenotype IURD analyses in FinnGen. P-values are highlighted as green when GWS, and yellow when not GWS but smaller than 0.01. *Strep throat was analyzed as a quantitative trait by Tian et al

## Supplementary Data 7

Colocalization results from eCAVIAR-powered in-house pipeline for single-phenotype IURD GWASs (pheno1) and eQTL data from relevant databases (SOURCE). Colocalization was considered significant if posterior probability (CLPP) was >20 % or posterior agreement (CLPA) was >50 %.

## Supplementary Data 8

Genes identified as associated with particular IURDs using MAGMA gene-based analysis.

## Supplementary Data 9

Colocalization results from eCAVIAR-powered in-house pipeline for single-phenotype IURD GWASs (column A) and non-IURD phenotypes in FinnGen main analyses. Colocalization was considered significant if posterior probability (CLPP) was >20 % or posterior agreement (CLPA) was >50 %.

## Supplementary Data 10

Epidemiological overlap between IURD cases and controls in the FinnGen R6 dataset. Each row shows the number of individuals with the row's phenotype (N), number of individuals with both row and column phenotype, and the proportion of cases of row phenotype that are also cases in the column phenotype. For instance, B2 denotes the number of individuals with vasomotor and allergic rhinitis (VAR; 8,975); cell E2 denotes the number of individuals with VAR who also have chronic rhinosinusitis (CRS; 1,415); and cell M2 denotes the number of participants with VAR who also have CRS (1,415 / 8,975 = 15.8 %). The diagonals of the overlap matrices separately denotes the number (bold) and fraction (white on black) of 'unique' cases, i.e. cases with no other IURD, such that e.g. C2 denotes the number of VAR cases with no other IURD (4,753), making 53 % (cell K2) of all VAR cases. In this way, we can see that the IURD with the highest level of comorbidity is nasal polyps (NP; only 34 % unique cases). Row 10 denotes the total proportion of FinnGen R6 individuals with the column phenotype. In other words, there roughly similar levels of NP in FinnGen in general (1.5 %) as there are NP cases among chronic diseases of tonsils and adenoids (CDTA; 1.6 % have also nasal polyps). NOTE: This table enumerates the absolute enrichments within FinnGen, and does not test for actual association between these diseases

## Supplementary Data 11

UKB read codes used in replication analysis. Cases from UKB for each replicated endpoint ("Endpoint") were chosen based on existence of a denoted read code ("Read codes"), with total cases denoted in "Cases". "Controls" represents the total number of post-QC UKB participants with no instance of any of the read codes, i.e. the same control group was used for all analyses as in the FinnGen analysis.

## Supplementary Data 12

FinnGen cohort list of author names, affiliations, and contributions to the FinnGen project.
